# Mechanical Properties of Mortars Reinforced with Amazon Rainforest Natural Fibers

**DOI:** 10.3390/ma14010155

**Published:** 2020-12-31

**Authors:** Régis Pamponet da Fonseca, Janaíde Cavalcante Rocha, Malik Cheriaf

**Affiliations:** 1 Post-Graduate Program in Civil Engineering, Federal University of Santa Catarina, Laboratory of Waste Valorization and Sustainable Materials (ValoRes), CEP 88040-900 Florianópolis, SC, Brazil; regis_pamponet@hotmail.com; 2 Department of Civil Engineering, Federal University of Santa Catarina, Laboratory of Waste Valorization and Sustainable Materials (ValoRes), CEP 88040-900 Florianópolis, SC, Brazil; malik.cheriaf@gmail.com

**Keywords:** amazon rainforest natural fibers, fiber reinforced cement mortars, mechanical properties, physical-chemical treatments

## Abstract

The addition of natural fibers used as reinforcement has great appeal in the construction materials industry since natural fibers are cheaper, biodegradable, and easily available. In this work, we analyzed the feasibility of using the fibers of piassava, tucum palm, razor grass, and jute from the Amazon rainforest as reinforcement in mortars, exploiting the mechanical properties of compressive and flexural strength of samples with 1.5%, 3.0%, and 4.5% mass addition of the composite binder (50% Portland cement + 40% metakaolin + 10% fly ash). The mortars were reinforced with untreated (natural) and treated (hot water treatment, hornification, 8% NaOH solution, and hybridization) fibers, submitted to two types of curing (submerged in water, and inflated with CO_2_ in a pressurized autoclave) for 28 days. Mortars without fibers were used as a reference. For the durability study, the samples were submitted to 20 drying/wetting cycles. The fibers improved the flexural strength of the mortars and prevented the abrupt rupture of the samples, in contrast to the fragile behavior of the reference samples. The autoclave cure increased the compressive strength of the piassava and tucum palm samples with 4.5% of fibers.

## 1. Introduction

Vegetable fibers have been used to reinforce building materials since the beginning of human civilization, for the construction of tents and walls. Vegetable fibers, mixed with earth and cementitious materials, were used to improve the mechanical strength of these materials; however, this use was determined empirically [1,2].

Mineral fibers (asbestos) are present in ceramic artifacts from the Neolithic period. In the industrial revolution, this mineral fiber was the most used in the world. The asbestos fibers were used to cool boilers, replacing silicate cotton; thereafter, asbestos was widely used in developing countries, such as Brazil, to manufacture fiber cement roofs due to its remarkably high mechanical performance and durability and it is noted significant thermal insulation efficiency [3,4].

Two other mineral fibers have been used to fabricate fibers composites (basalt and glass fiber). The production of basalt and glass fibers are similar. Crushed basalt rock is the only raw material required for manufacturing the first one and glass fibers is produced from various components. Basalt fibers were only developed in recent decades and require less energy to be produced. Their strength and modulus properties are situated among those offered by glass fibers. Although current research shows that the structural behavior, including long-term deflections due to creep and cyclical loading, is like glass fiber, internationally recognized code authorities have yet to acknowledge basalt in their codes. The disadvantage of basalt fiber is that it breakable and low in withstanding bending force; in the production of the fiber, basalt rock is melted over 1500 °C, consuming a lot of energy [5].

Synthetic fibers such as polyvinyl alcohol (PVA) and polypropylene replaced asbestos fibers due to legal and health problems. PVA fibers have high mechanical strength, are resistant in an alkaline environment, and provide strong adhesion to the cement matrix; however, they are expensive. Compared to them, polypropylene fibers have a low tensile strength and modulus of elasticity and provide low adhesion to the cement matrix [6,7].

Over the years, research has been developed to fabricate new composites materials to the civil construction field. Steel bars in reinforced concrete can deteriorate by corrosion. Al-Rubaye et al. [8] developed a hollow composite reinforcing system (CRS) with Glass Fiber Reinforced Polymer (GFRP) bars to investigate the flexural behavior of concrete slabs, as an alternative to the steel bars in the concrete slabs. The hollow system was more compatible with GFRP bars than steel bars due to their similar modulus of elasticity.

When reinforced concrete structures suffer deterioration problems such as corrosion, it is common to use the same materials to repair the structures and the damage rehabilitation cannot work as imagined. Mohammed et al. [9] developed a prefabricated FRP (fiber reinforced polymer) jacket used to rehabilitate a reinforced concrete column structure simulating corrosion damage. As a result, the FRP jacket stabilized the damaged column by reducing the eccentricity effect of the corroded steel and restored the axial stiffness and the strength capacity.

The use of vegetable fibers, with characteristics similar or superior to mineral and synthetic fibers, becomes viable for the production of sustainable materials, since these fibers come from renewable, biodegradable, and widely available sources, and their cost is relatively low. Additionally, they have a good ability to absorb mechanical impacts [10,11].

Brazil is one of the largest natural fiber producing countries in the world, with countless species of plants and their respective fibers from the Amazon rainforest [12]. Vegetable fibers from plants in the Amazon rainforest began to be used for several industries in the Manaus Free Trade Zone. The waste from these industries is approximately 1 ton/day, according to the development study of an integrated solution related to the management of industrial waste in the industrial hub of the city of Manaus [13].

The fibers of the Amazon piassava species (*Leopoldinia piassaba*), the tucum palm (*Astrocaryum chambira Burret*), and razor grass (*Echinochloa polystachya*) can potentially be used in the production of cementitious composites, but there are no studies on the influence of the physical and chemical properties of these fibers on the mechanical properties of cementitious composites. Due to the large amount of research on the physicochemical properties of jute fiber, this fiber was used in the present study for comparative purposes only among the piassava (*Leopoldinia piassaba*), tucum palm (*Astrocaryum chambira Burret*), and razor grass (*Echinochloa polystachya*) fibers.

To ensure the dimensional stability, strength, and durability of plant fibers, the natural fibers must be specially treated by physical-chemical treatments [14]. The incorporating of supplementary cementitious materials (SCMs) must improve the susceptibility to portlandite. [15,16].

Generally, a single treatment at each time is applied to the fibers. When hybridization is used as a treatment, it is possible to remove surface impurities from the fiber cell wall; under chemical treatments, the polymer constituents of the fibers are transformed and become potentially solubilized in water. Since the high moisture absorption is a great problem for natural fiber composites, especially in in outdoor structures, the hornification treatment causes shrinkage of the fibers and is an irreversible effect on lignocellulosic fibers when they are subjected to dry/wet cycles. Due the formation of hydrogen bonds in cellulose and does not modify the resistance of the fibers. This effect is quantified as the reduced percentage and the ability to absorb water and moisture.

The vegetable fibers present high-water absorption, and this property can be an obstacle for incorporation in cementitious composites. The high-water absorption of the fibers is responsible for the weakening of the fiber/matrix bond and the interfacial transition zone (ITZ) due to the increased porosity [17].

In the case of mortars reinforced with vegetable fibers, the portlandite presence in the pulp is synonymous of ITZ fragility; therefore, the use of CO_2_ curing procedures can contribute to its densification, allied with the application of natural fiber treatments with the purpose of promoting roughness, increasing its surface area, and decreasing water absorption [15,16,18].

The objective of this research was to evaluate the use of natural fibers, as an alternative to reinforce cement-based composite materials, through the proposal of alternative treatments applied to natural fibers. For the manufacture of mortars, supplementary cementing materials (SCMs) were incorporated as well as the use of curing in CO_2_ autoclave, both with the purpose of improving the mechanical properties and durability of fiber-cement composites.

The novelty of this research is the use of fibers from the Amazon that have never been used (such as tucum and razor grass) and simple treatments (such as the hybridization treatment) combined with the cure with autoclaved CO_2_ that can provide a densification of the matrix. The result of this study will benefit researchers and end users of remote areas, with tropical forests, where there are numerous plant residues, encouraging them to seek alternatives and easy-to-use methods so that fiber cement with natural fibers has the appropriate efficiency for the manufacture of functional materials for engineering.

## 2. Materials and Methods

### 2.1. Materials

The cement used in the present study was Portland CPII-F 40, according to Brazilian standard NBR 16697 [19] corresponding to type II (ASTM C150 [20]), without any pozzolanic materials in its composition. The fly ash is from pulverized coal mineral produced in thermoelectric plants of a Brazilian company involved in the cellulose industry. The fly ash was dry sieved to obtain particles of less than 75 µm. The metakaolin used was HP ULTRA, a mineral admixture provided by a Brazilian metakaolin company. The chemical compositions and physical properties of cement fly ash and metakaolin are presented in Table 1. Chemical composition was determined by energy dispersive X-ray fluorescence spectrometer EDX 7000 Hs (Shimadzu, Tokyo, Japan), in terms of total oxide (wt.%).

To produce mortars, natural sand was used as a fine aggregate for the composites. The fineness, specific gravity, and natural moisture content of this sand were respectively 1.90, 2.65 g/cm^3^, and 7.90%. A superplasticizer additive (SP) based on sodium polycarboxylate was used to improve the fluidity of the fresh mixtures.

The vegetable fibers used in the study come from the “Dog’s head” region in the far northeast of the state of Amazonas, in the northern area of Brazil.

The tucum palm fibers have lengths between 2.00 m and 8.00 m and 0.08 mm of thickness (does not have a circular shape), the razor grass fibers have lengths between 0.5 m and 0.75 m with 1.00 mm of diameter, both coming from the leaves of the plants. Jute fibers have a length of 1.00 m to 4.00 m with 1.08 mm of diameter, piassava fibers have lengths ranging from 0.60 m to 2.00 m with 0.8 mm of diameter, both coming from the stems of the plants.

The Young’s modulus of tucum palm, razor grass, jute and piassava fibers were 4.03 GPa, 70 GPa, 10 GPa and 1.20 GPa; respectively. All nature fibers presented an Elongation at failure (%) of the 1.5%, 0.5%, 1.9% and 17.8%; respectively to the tucum palm, razor grass, jute and piassava fibers.

**Figure 1:**
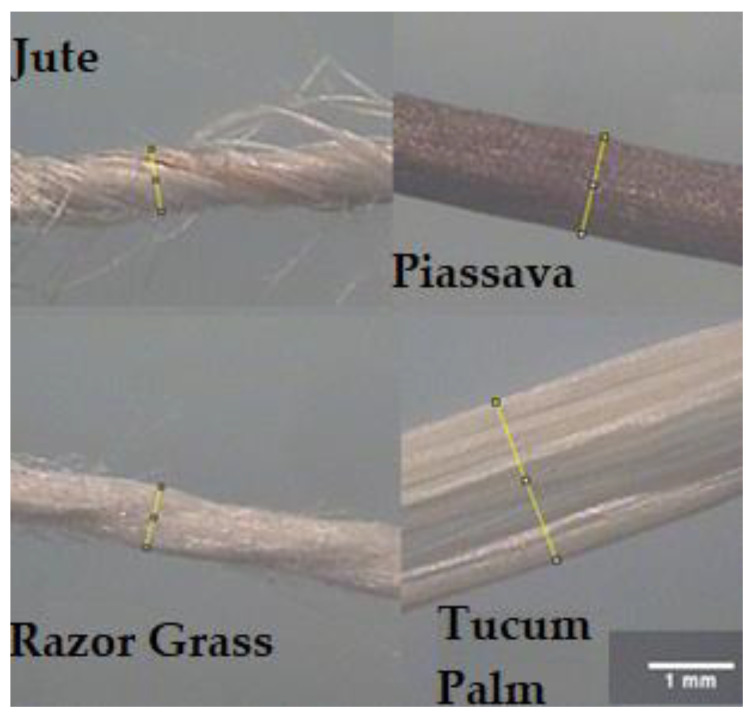
Image of untreated fibers. Fibers: Jute (from the stems of the plants), Piassava (from the stems of the plants), Razor Grass (from the leaves of the plants) and Tucum Palm (from the leaves of the plants). (All images with 20× enlargement photos).

Figure 1 shows 20× enlargement photos of untreated fibers observed under a Binocular Stereoscopic Microscope model Stemi 2000-C from Zeiss (Oberkochen, Germany). The image enlargement capacity of microscope is from 6.5× to 50×.

Natural fibers were sampled for chemical analysis following the Standards of the Pulp and Paper Industry Technical Association (TAPPI), based on solvent extractions to quantify cellulose and alpha cellulose [21], extractives [22], lignin [23], and ash content [24]. Therefore, they were chemically characterized to quantify cellulose, extractives, lignin, and ash (Table 2).

Lignin and hemicellulose are amorphous polymers that give less tensile strength to the fibers, and their dissolution is one of the main reasons for fiber deterioration in cementitious matrices. Fibers with a high cellulose content usually present superior tensile strength since this is the most resistant and crystalline component of its composition [25].

Razor grass fibers had a low lignin content and the highest cellulose content (holocellulose and alpha cellulose), while piassava fibers had the highest lignin content. Such results influence both the tensile strength of the fibers and the compressive strength of the mortars, since lignin weakens the fiber/matrix bond [14,15].

### 2.2. Methods

#### 2.2.1. Treatments of the Fibers

To improve the physical and mechanical properties of the fibers, four types of treatments were applied: fibers washed in hot water, hornification, sodium hydroxide treatment, and hybridization. After the treatment, all the fibers were cut to 15 mm in length. In addition, to work as reinforcement, fibers agglomerations should not occur during mixing. The length of 15 mm is classified as a ‘‘short fiber”. This length is reported in the literature [7,12] and it was chosen to provide homogeneous dispersion of fibers in the matrix without agglomerate. If the length of the fiber is too long, it can break inside the composite and not gain strength, also the length is appropriate for larger samples or structures. The natural fibers were rinsed in water (for surface dirt removal) and dried in an oven at a temperature of 60 °C for 24 h.

The treatment of washing in hot water was carried out according to [7,26]. The fibers were placed in hot water at a temperature of 100 °C for 2 h, then they were placed into a heating chamber at a temperature of 60 °C and dried for 24 h, cooled for 30 min, and then sealed in plastic bags for testing.

The hornification treatment process consisted of the application of 10 wetting/drying cycles, with the procedure similar to [17,27,28], where one cycle consisted of the following procedures: fibers were submerged for 9.00 h in water at a temperature of 23 °C; then excess water was drained for 30 min through a sieve; the fibers were dried for 14 h in the heating chamber at 60 ± 5 °C; and finally, they had 30 min cooling at room temperature, totaling a 24-h cycle.

For the treatment with NaOH (Neon Commercial, Suzano, Brazil), the fibers were placed in the sodium hydroxide solution (2 mol/L), with the ratio between the fibers’ weight and solution used being 1:20. This was mixed homogeneously for 5 min and then rested for 1 h. After this period, the fibers were placed in distilled water for 3 min, then were removed from the water and placed in the glacial acetic acid solution (1%) to neutralize for a 10-min period. Then the fibers were washed in running tap water for 5 min and rinsed in distilled water until the pH = 7 (2 min).

The hybridization process of treatment consisted of the following four stages: the fibers were washed in hot water; the hornification cycles process; treatment with NaOH; treatment with the application of 5% hydrogen peroxide solution, with 1:20 ratio between the fibers’ weight and the solution. In this latter treatment, the fibers were mixed homogeneously for 5 min in the peroxide solution and left to stand for 3 h; then, the fibers were placed in distilled water for 3 min, and then were washed in running tap water for 5 min, then rinsed in distilled water until the pH = 7 (2 min).

After the treatments, the fibers were evaluated by determining the crystallinity index by X-ray diffraction (XRD) and measuring their tensile strength.

#### 2.2.2. Water Absorption of the Fibers

The test for the water absorption capacity of untreated and treated fibers was performed as follows: the fibers were cut to a length of 15 mm long with scissors, dried at a temperature of 60 ± 5 °C, and cooled for 30 min until the difference in mass was less than 0.1 g between the two consecutive measurements that were made. After this procedure, 2.00 g of dry fiber (Wdf) were weighed and placed in a tea infusion ball (to help the fibers sink) and covered with water for 24 h. After this period, the remaining water from the fibers was removed by sieving (1 min), then the fibers were wrapped in absorbent paper for 1 min (this condition was Wssd = saturated and dry surface) and their weight was immediately determined. The absorption of water by the fibers was calculated in percentage of mass, according to the following Equation (1):

(1) $$\mathrm{WA}\;\left(\%\right)=\;\frac{\mathrm{Wssd}-\mathrm{Wdf}}{\mathrm{Wdf}}\;\times \;100$$

#### 2.2.3. Crystallinity Index (CI %)

To measure the crystallinity index of the fibers before and after the treatments, the XRD technique was used. The crystallinity index was determined according to the empirical method suggested by Segal et al. [29]. This method consists of the calculation of the cellulose crystallinity index (CI) according to the following Equation (2):

(2) $$\mathrm{CI}=\frac{I_{002}-I_{\mathrm{am}}}{I_{002}}$$

where: I_002_ corresponds to the maximum intensity of the diffraction peak, related to the crystalline part of the cellulose in plane (002) and I_am_ refers to the intensity of the background dispersion (amorphous part of the sample).

Two fiber samples were cut by hand to a length of 1 cm and crushed in a knife mill (Wiley Model 4 from Thomas Scientific, Swedesboro, NJ, USA) for 10 min. The fibers were then sieved in a 60-mesh sieve (0.25 mm) and dried at a temperature of 60 °C for 24 h in the heating chamber.

XDR analyses were performed using a desktop Rigaku Miniflex II (Tokyo, Japan) at an angular velocity of 0.02° per second, and Bragg angle measurement intervals (2θ) from 5° to 60°, and the analyses were conducted with (Cu radiation) kα = 1.5418 Å.

#### 2.2.4. Direct Tensile Strength of the Fibers

The direct tensile strength test of the fibers was measured according to ASTM C1557 [30] using a universal Instron 5569 machine (Norwood, MA, USA). The test speed was 0.3 mm/min with a load cell of 1 kN. The 12 fiber samples for each treatment were conditioned at room temperature (23 °C) and prepared as recommended by the ASTM C1557 standard. The effective cross-sectional area was assumed to be circular. The length of the fibers was 50 mm. The direct tensile strength was calculated by dividing the breaking force (F) in Newtons by the cross-section area (A) in mm^2^.

#### 2.2.5. Thermogravimetry of Pastes

To identify the reduction of calcium hydroxide promoted by metakaolin (MK) and fly ash (FA), pastes were produced with a water-to-binder (OPC + MK + FA) ratio of 0.4. OPC was used as a reference (control) and replaced 50% (wt.%) of the Portland cement by a binary blend formed by 10%:40%, 20%:30% and 40%:10% (MK:FA), by mass. The paste study was performed in order to establish the optimal blend, i.e., the most effective for reducing calcium hydroxide or Portlandite (CH). All pastes were produced with a ratio of 1:1 (OPC: MK + FA) and a water-to-binder ratio of 0.4. This ratio was chosen based on the literature [31,32,33]. Table 3 summarizes the compositional paste mixes studied.

The sample paste (cylinder with 1:2 ratio diameter of 2 cm by 4 cm length) was tested by compressive strength testing at the age of 28 days. The fragments were used to assess thermogravimetric performance. The thermogravimetric analysis was performed using a TA Instruments Q600 SDT Thermo Gravimetric Analyzer (New Castle, DE, USA) at a heating rate of 20 °C/min from 20 °C to 1000 °C in a N_2_-atmosphere, to measure the CH content. The CH content was determined from the mass loss between 400 °C and 500 °C from Equation (3).

(3) $$\mathrm{CH}=\frac{W_{400}-W_{500}}{W_{500}}\;\times \;\frac{M_{\left(\mathrm{Ca}\left(\mathrm{OH}\right)2\right)}}{M_{H2O}}$$

where:

W_400_ = sample mass at 400 °C.
W_500_ = sample mass at 500 °C.
$\frac{M_{\left(\mathrm{Ca}\left(\mathrm{OH}\right)2\right)}}{M_{H2O}}\;$ = ratio between CH molar mass and H_2_O molar mass = 4.11

To evaluate the fibers in Portland cement-based composites, mortars with a mass ratio of 1:2 (binder-to-sand) and water to cement (w/c = 0.6) were cast into a prismatic mold with dimensions 40 mm × 40 mm × 160 mm, according to NBR 7215 [34]. After being demolded, the samples were cured until testing. The samples were prepared for submission to two series of cures: in water and in the CO_2_-autoclave. For each cure, the number of samples was (N = 3). For this purpose, the mix (50 OPC + 40 MK + 10 FA) with 1.5%, 3.0% and 4.5% of fibers was used to study the mortar and its performance compared with a reference mortar (OPC + MK + FA) without fibers.

It should be noted that the fibers were previously saturated for 24 h before molding, with the amount of water added to the mixture being corrected, i.e., decreased, to take into account the amount of water already absorbed by the fibers. After saturation, three proportions (1.5%, 3.0%, and 4.5%) of fibers were mixed with the mortar. The mixes were made using a planetary mixer. First the binder materials (OPC + MK + FA) were mixed for 1 min at slow speed, then 80% of water and 80% of admixture were added and mixed for a further 1 min at slow speed, then dry sand was added and mixed for a further 1 min at the same speed. Finally, the last 20% of water and 20% of admixtures were added, the wet fibers were randomly added, and mixed for 3 min at medium speed.

#### 2.2.6. Curing Methods

The following two procedures for curing mortars were used: curing in water and curing in an autoclave with CO_2_ inflation.

The cure in water consisted of leaving the composites submerged in water for 28 days at 23 °C; after 28 days they were dried in a heating chamber for three days at 50 °C. The autoclave cure consisted of placing the samples inside the autoclave, then filling it with 22 L of tap water. With a rubber hose, CO_2_ was inflated in the water for 7 min at a flow rate of 6 L/min, the pH was verified through tapes (values between 5 and 6). The autoclave was then closed and switched on until the temperature was sufficient to generate a pressure of 0.05 MPa. After reaching this pressure, it was left on for 1 h each day during the 28 days of the curing period.

#### 2.2.7. Compressive and Flexural Strength

Mechanical tests of strengths were performed on the mortars (flexural and compression) and pastes (compression) both at 28 days, and after 20 drying/wetting cycles (durability) for the mortars. For the pastes, 3 cylindrical specimens of 20 mm × 40 mm [34] were molded and the loading rate performed was (0.25 ± 0.05) MPa/s. And for the mortars, 3 prismatic samples of 40 mm × 40 mm × 160 mm were used, according to the NBR 13279 [35]. For the flexural test, the loading rate performed was (50 ± 10) N/s and for the compression test the loading rate performed was (500 ± 50) N/s until the sample collapse [35].

## 3. Results and Discussion

### 3.1. Water Absorption of Fibers

**Figure 2:**
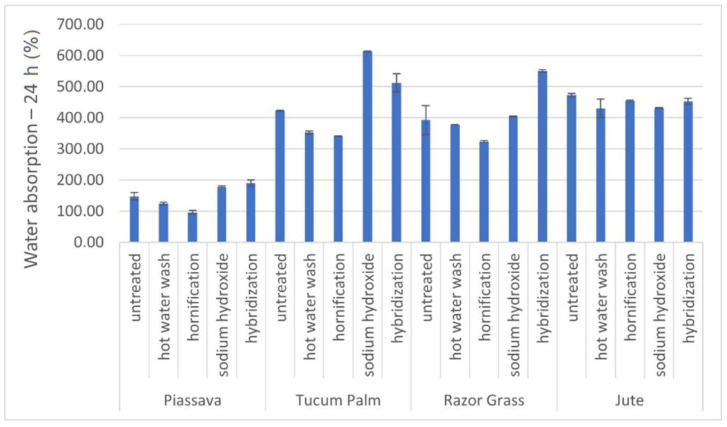
Water absorption—24 h of treated fibers compared with untreated. Four fibers: piassava, tucum palm, razor grass, and jute. (N = 3).

Figure 2 shows the results of water absorption of untreated and treated fibers after 24 h of immersion. Water absorption increased for tucum and razor grass fibers through chemical treatments (NaOH and hybridization). The fibers from shrubs and palm trees present great water absorption, usually above 400% by mass [33].

The chemical treatments removed dirt and part of the lignin impregnated in the fibrils, causing greater water absorption by the fibers, since it exposed OH groups (hydroxyls) belonging to cellulose. The increase in the amount of cellulose improves the mechanical strength of the fiber. Bleaching makes the fibers more uniform, due to the removal of some extracellular materials; the degree of crystallinity increases (observed in tucum fibers, razor grass, and jute) and the fibers acquire a whiter color. Water absorption is increased due to the removal of hydrophobic substances exposing hydrophilic sites [34,35,36].

### 3.2. Crystallinity Index

**Figure 3:**
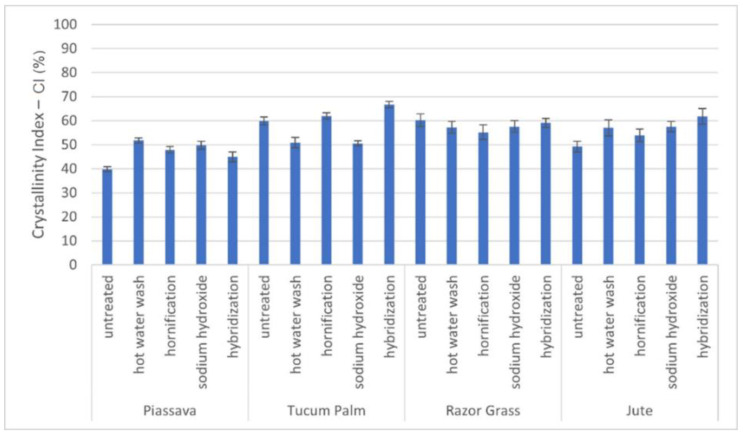
Results of crystallinity index—CI % of untreated and treated fibers. Four fibers: piassava, tucum palm, razor grass, and jute.

**Figure 4:**
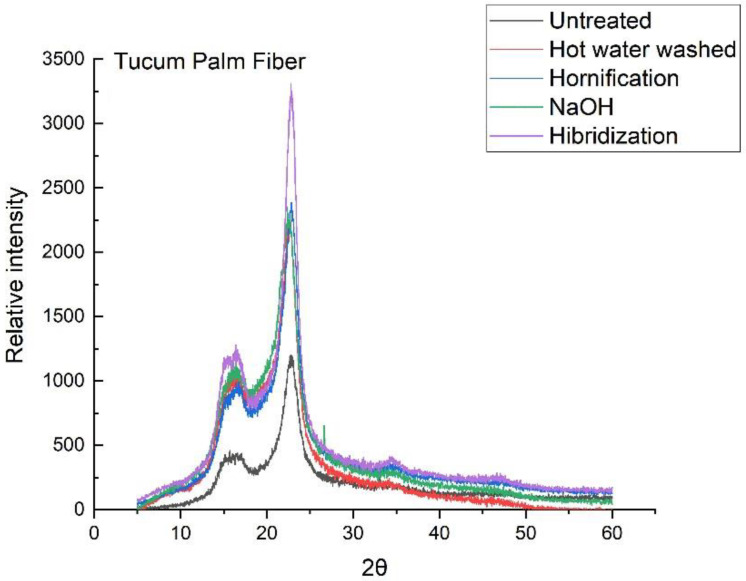
X-ray diffractograms of the untreated and treated fibers of the tucum palm. Different conditions: untreated (black), hot water washed (red), hornification treatment (blue), NaOH (green), and hybridization (purple).

According to the graph in Figure 3, the highest crystallinity index of piassava fiber was obtained with the hot water treatment (51.80%), and for tucum fiber by the application of the hybridization treatment (66.73%), where the highest crystalline peak in the plane (002) is observed (Figure 4). This possibly led to the increased packaging of cellulose chains and the transformation of cellulose type I into cellulose type II, the latter being thermodynamically more stable and providing the increased tensile strength of the fiber [37,38,39,40].

On the other hand, for the razor grass fibers, the result of the crystallization indexes of the treated fibers is slightly lower than that of the untreated fiber on average (57.86%), due to the high percentage of holocellulose present in natural (untreated) fiber (95.30%) which did not become type II, even after alkali treatments.

### 3.3. Direct Tensile Strength of Fibers

**Figure 5:**
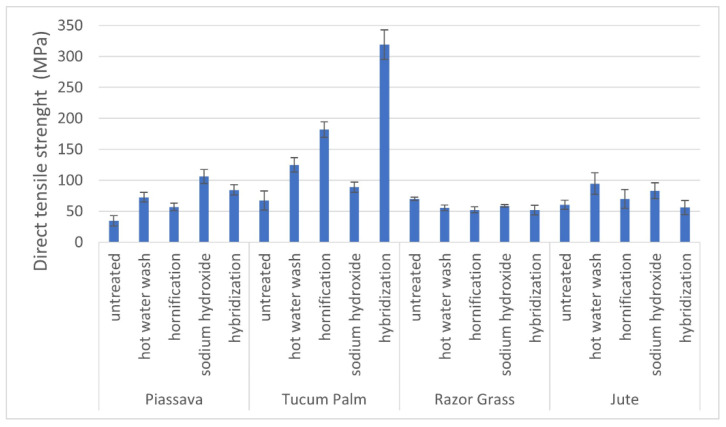
Direct tensile strength of untreated and treated fibers. Four fibers: piassava, tucum palm, razor grass, and jute.

Figure 5 shows the results of the direct tensile strength of treated and untreated fibers. The hybridization treatment of tucum fibers had the highest tensile strength result (318.81 MPa) as well as the highest crystallinity index (66.73%), indicating that the treatment possibly removed part of the small amount of hemicellulose present in the untreated fiber (1.38%) packaging the cellulose chains.

However, for the razor grass fibers, none of the treatments applied improved the tensile strength; all results were inferior to those of the untreated fiber, close to the findings of the authors which stated [33,41,42] that the crystallinity index of the fiber is not the only parameter which serves to establish or identify its mechanical performance. Moreover, the analysis of other parameters, such as the angle and orientation of microfibrils, the number of cellulose crystals that compose the fiber, the length and width of the unit cells also shows that they exert influence on the mechanical performance. It can be observed that the razor grass fiber presented a high alpha cellulose index (86.85%) where the treatments could not rearrange the fibrils in the direction of axial traction deformation, so there was no contribution to the tensile strength.

### 3.4. Metakaolin (MK) and Fly Ash (FA) Content Determination

**Figure 6:**
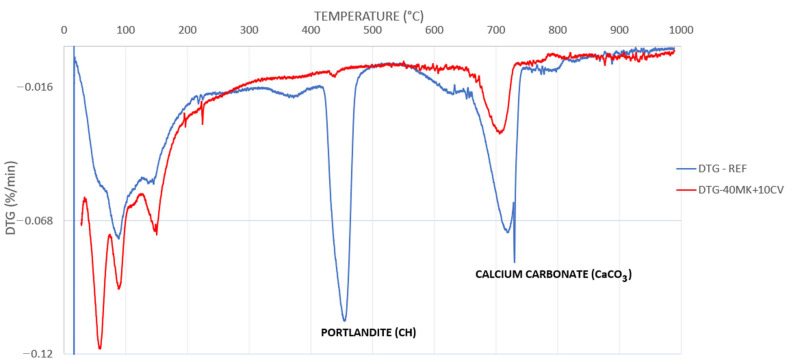
(DTG) curves of cement and blended paste (40 MK + 10 CV).

The derivative thermogravimetry (DTG) curves of the samples of the investigated pastes after 28 days are shown in Figure 6. The decrease in Portlandite peaks (CH) is observed between 400 °C and 500 °C due to the substitution by MK and FA. This reduction is due to the reaction of MK and FA with CH to form more C–S–H in the pastes.

Table 4 shows the CH percentages calculated for the (OPC + MK + FA) pastes. The cement pastes with 40% MK and 10% FA showed the lowest percentage of CH (3.84%) and the highest compressive strength (42.12 MPa), when compared with the reference paste (REF-OPC). Due to low CH and a good mechanical performance (42.12 MPa), the paste formed by 50% of OPC + 40 MK + 10 FA was fixed as a binder to produce the mortars.

### 3.5. Chosen Fiber Treatment for Reinforced Mortars

After treatment, four fibers were selected based on the crystallinity index (CI) and mechanical performance. For the mortars with piassava and tucum fibers, the hot water and hybridization treatments were chosen, respectively, as both had the highest crystallinity indexes (CI %), and for the tucum fiber the highest tensile strength (318.81 MPa).

For the razor grass fiber, the hornification treatment was chosen, since the fiber resulted in a decrease in water absorption, and finally, for the jute fiber, the NaOH treatment was chosen, since the fiber did not present additional degradation due to the alkaline treatment.

The treatments were chosen to try to combine one or more results, such as higher results of tensile strength, higher crystallinity index, and lower water absorption values of the fibers. These results are believed to lead to high mortar strengths.

### 3.6. Compressive Strength

**Figure 7:**
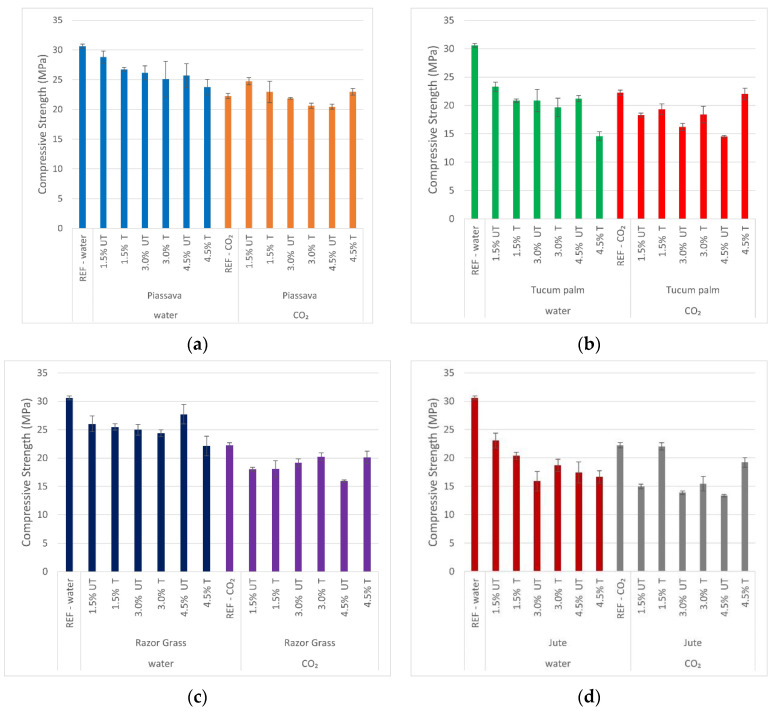
Mortars’ compressive strength at 28 days obtained with samples cured with water and CO_2_: (**a**) 1.5%; 3.0%; 4.5% piassava mortars; (**b**) 1.5%; 3.0%; 4.5% tucum palm mortars; (**c**) 1.5%; 3.0%; 4.5% razor grass mortars; (**d**) 1.5%; 3.0%; 4.5% razor grass mortars; REF—specimen without fiber; UT—untreated fiber mortar; T—treated fiber mortar; water and CO_2_—cured methods.

Figure 7 shows the compressive strength of mortars with treated and untreated fibers, with two types of curing: water curing, and with CO_2_ autoclaved. It can be observed that the compressive strength of the reference sample without fibers which had been cured under CO_2_-autoclaved, was lower than that of the wet-cured sample. This fact is due to the change in the hydration chemistry of the cement.

According to the authors [43,44], the amount of SiO_2_ present in binder materials must be sufficient for the conversion of C–S–H into tobermorite (C_5_S_6_H_5_). The CaO/SiO_2_ ratio must be between 0.88 and 1.36 and the temperature must be above 100 °C, to be suitable for the formation of tobermorite.

In the present study, the amount of metakaolin (43.90%) and fly ash (61.18%), together with the autoclaved chamber temperature (below 100 °C) and pressure (0.15 MPa), was not enough for this conversion and it was one of the causes of the reduction of compressive strength.

However, despite the decrease in compressive strength due to the autoclaved cure, Table 5 presents the relationship between the crystallinity index and the compressive strength of mortars. It can be observed that the mortars cured in the autoclave, with treated fibers of tucum and jute, presented better compressive strength as the percentage of fiber increased (4.5%), evidencing the efficacy of the treatments to which these fibers were submitted.

**Figure 8:**
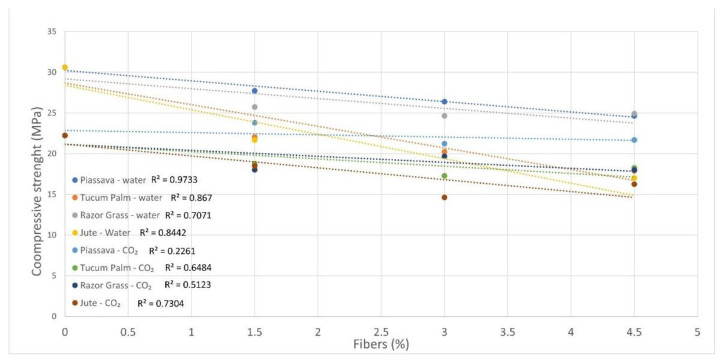
Correlation between compressive strength (28 days) versus fibers’ content. R^2^ values are presented on the graphs.

The slight increase in the strength of mortars with 4.5% of tucum palm fibers (Figure 7b) treated by hybridization, and jute fibers treated with NaOH solution (Figure 7d), was improved through the hydrophilicity presented by these fibers, and the removal of hydrophobic substances, allied to the increase in the roughness of their surfaces. For this reason, it is noted that the coefficient of determination (R^2^) of mortar samples with the autoclaved cure decreases, and with this, the existing linear relation is lost. The higher the percentage of fiber is, the lower is the compressive strength of the mortars (Figure 8).

### 3.7. Flexural Strenght

**Figure 9:**
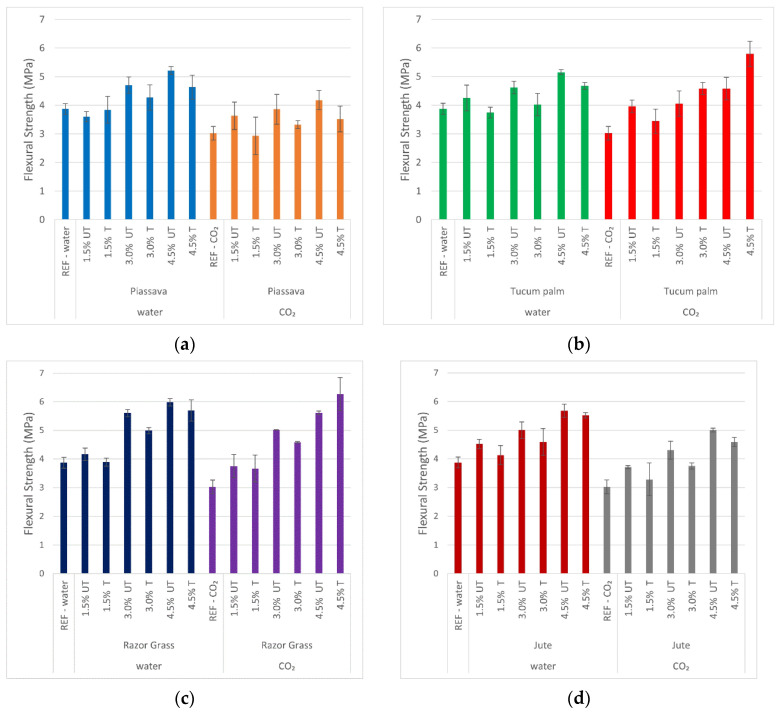
Flexural strength of mortars at 28 days obtained with samples cured with water and CO_2_: (**a**) 1.5%; 3.0%; 4.5% piassava mortars; (**b**) 1.5%; 3.0%; 4.5% tucum palm mortars; (**c**) 1.5%; 3.0%; 4.5% razor grass mortars; (**d**) 1.5%; 3.0%; 4.5% razor grass mortars; REF—specimen without fiber; UT—untreated fiber mortar; T—treated fiber mortar; water and CO_2_—cured methods.

The flexural strength of treated autoclaved fiber samples was lower than those samples cured in water. However, for samples with tucum palm (5.73 MPa) and razor grass (6.19 MPa) fibers, the results were slightly higher than the samples cured in water, respectively (4.64 MPa and 6.07 MPa) Figure 9.

These results suggested that the treatments applied (hybridization and hornification) to these fibers promoted the best compatibility of connection between the fiber and matrix, along with the autoclave cure procedure. The alkaline hybridization treatment led to increased ductility and improved mechanical performance of samples with tucum palm fibers, since it reorganized the cellulose chains and divided the fiber into many more fibrils, which in turn raised the mechanical property of the direct tensile strength of the fiber from 67.2 MPa to 318.81 MPa and increased the CI % crystallinity index from 59.84% to 66.73%.

The hornification treatment of the razor grass fiber decreased the water absorption of the fibers, and consequently, the mixture had more free water to moisturize the pulp, and the greater amount of fibers (4.5%) resulted in the increase of fiber/matrix adhesion. This led to an increase in flexural strength of 19.20% for treated mortars cured in the autoclave with inflated CO_2_ [17,45].

**Figure 10:**
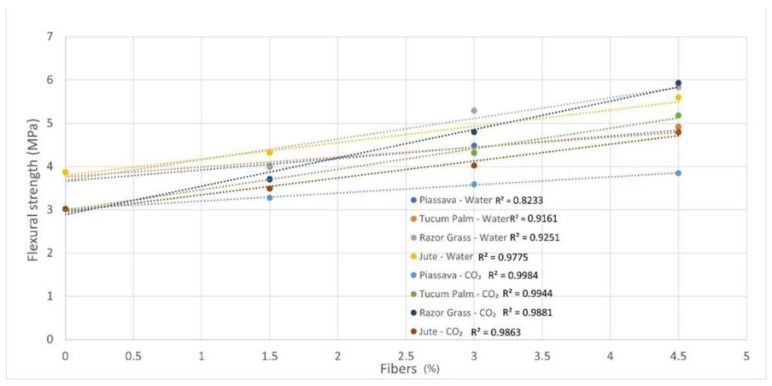
Flexural strength of mortars (28 days) obtained from water and CO_2_ curing versus fibers content. R^2^ values are presented on the graphs.

The coefficient of determination (R^2^) of the flexural strength of mortar samples is higher for samples cured with CO_2_, compared with those for samples cured in water as can be seen in Figure 10—a result which shows the effectiveness of the curing procedure for the fiber samples.

### 3.8. Durability Cycles

#### 3.8.1. Durability Cycles—Compressive Strength

**Figure 11:**
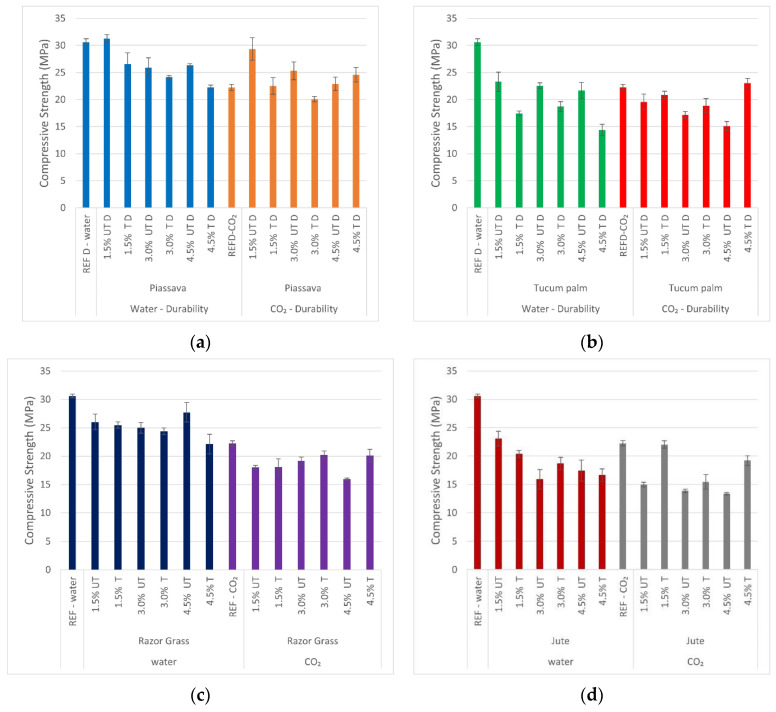
Durability cycles—compressive strength mortars: (**a**) 1.5%; 3.0%; 4.5% piassava mortars; (**b**) 1.5%; 3.0%; 4.5% tucum palm mortars; (**c**) 1.5%; 3.0%; 4.5% razor grass mortars; (**d**) 1.5%; 3.0%; 4.5% razor grass mortars; REFD—specimen without fiber after durability cycles; UTD—untreated fiber mortar after durability cycles; TD—treated fiber mortar after durability cycles; water and CO_2_—cured methods.

Figure 11 shows the compressive strength of fiber mortars after durability cycles. The piassava fiber (4.5%) samples increased from 22.23 to 24.59 MPa, and for the tucum palm (4.5%) samples the increase was a slight one from 22.23 to 23.02 MPa, with both cured in the CO_2_ autoclave. For the razor grass (4.5%) and jute (4.5%) samples, the compressive strength slightly decreased, to, 21.64 MPa and 20.19 MPa, respectively. Despite this small strength decrease, it can be observed that 20 cycles of wetting and drying and the curing procedure did not cause a large degradation of those samples, since the compressive strength was above 20 MPa, and this proves that the treatments used were effective.

#### 3.8.2. Durability Cycles—Flexural Strength

**Figure 12:**
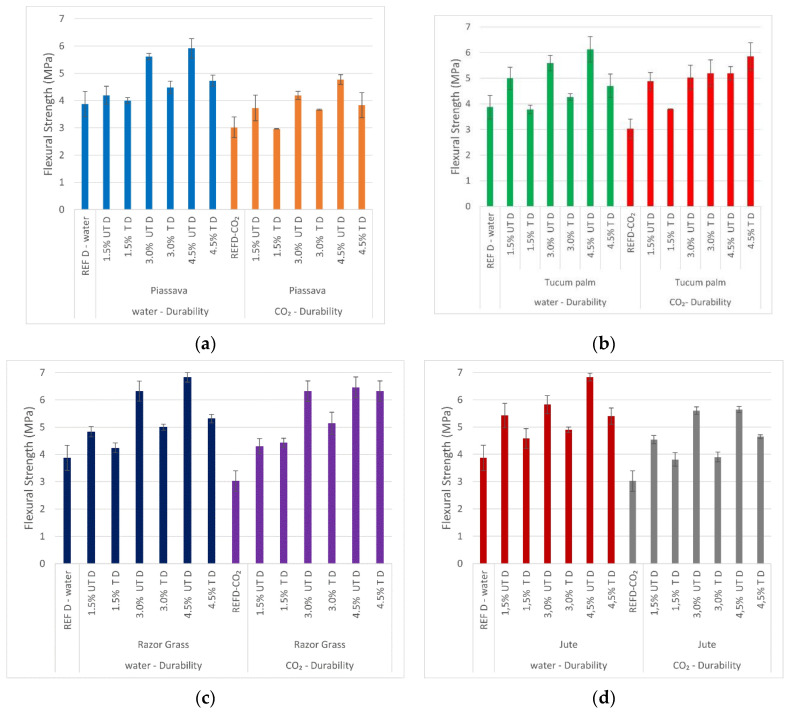
Durability cycles—flexural strength mortars: (**a**) 1.5%; 3.0%; 4.5% piassava mortars; (**b**) 1.5%; 3.0%; 4.5% tucum palm mortars; (**c**) 1.5%; 3.0%; 4.5% razor grass mortars; (**d**) 1.5%; 3.0%; 4.5% razor grass mortars; REFD—specimen without fiber after durability cycles; UTD—untreated fiber mortar after durability cycles; TD—treated fiber mortar after durability cycles; water and CO_2_—cured methods.

The flexural strength of the mortars with treated and untreated fibers after the durability cycles is shown in Figure 12. For composites with piassava and jute fibers, with the CO_2_ curing method and durability cycles, the flexural strength results were lower than those of composites subjected to water curing. This was due to the fact that the curing method is not so aggressive, from the point of view of humidity changes, and the fact that the hornification and hybridization treatments (applied to the tucum palm and razor grass fibers’ composites) have already modified the dimensional stability of those fibers; however, they preserved their ductility and reinforcement capacity, even in the severe conditions of CO_2_ curing.

According to Juarez et al. [2] and Claramunt et al. [17], the matrix with fly ash prevented the fiber deterioration caused by the humidity variations (in this research the deterioration caused by the cycles and CO_2_ curing method) and the hornification treatment had already changed the volumetric of the fibers.

## 4. Conclusions

The aim of study was to improve the characteristics of four natural fibers, from the Amazon forest (Piassava, tucum palm, razor grass and jute) to be used as an alternative for reinforcement in cement composite materials. Four alternative treatments were applied in natural fibers (hot water wash, hornification cycles, application of sodium hydroxide solution and hybridization treatment) combined with the incorporating of supplementary cementitious materials (SCMs) for mortars production under two cures types (CO_2_ autoclave and water cure) to densify the cementitious matrix.

From this experimental study, it can be deduced that the crystallinity index depends on the concentration of alkalis and the compression strength of mortars. The correlation of higher compression strength, higher CI %, was evidenced in mortars with tucum and jute fibers (hybridization and NaOH treatments, respectively) with curing in the autoclave. The treated fibers did not undergo further degradation due to the increases in temperature and pressure inside the autoclave.

In tucum fibers, the treatment improved the packaging of the cellulose chains and a greater number of connections were created between its microfibrils, resulting in the highest crystallinity indexes (66.73%) and greater tensile strength (318.81 MPa).

The performance of the hybridization treatment for the tucum fibers is evidenced by the results obtained by increasing the tensile strength and crystallinity indexes of the fiber. Therefore, it is a viable and attractive alternative treatment for its application since it modified the fibrillar structure through physical-chemical changes. In addition, piassava, razor-grass, and especially tucum fibers have physical properties in accordance with and superior to jute fiber, which can be used as a reinforcement in the development of sustainable building composite materials.

In addition, curing in an autoclave with CO_2_-saturated water, despite having shown significant reductions in the compressive strength of the composites, proved to be an interesting alternative, since it showed an improvement in the strength of the fiber and tucum and jute composites with the increased percentage of fiber (Table 5), and the durability cycles improved the mechanical properties and fiber-matrix bond, as shown in the compressive strength of treated piassava and tucum fibers which were CO_2_ cured.

The flexural strength (4.5%) of tucum palm and razor grass samples with the same curing procedure (CO_2_) preserved their ductility and reinforcing capabilities, reaching flexural strengths close to or higher than those with curing in water (a less aggressive curing method).

The failure mechanism of the composites is complex, involving flaws and cracks in the matrix. The structure of the fibers after treatments with surface protrusions, contributed to the minimum detachment of the fiber, and improved the performance of the composite during mechanical tests, after flexural bending limits, the composites continued to withstand load without suddenly break and with high deformation, especially for composites with piassava fibers. The results showed that above 3.0% of fibers the results of flexural strength are higher than those of the reference composite, in both types of cure and for all types of fiber.

In compression, the rupture occurred gradually, as the transfer bridges formed by the fibers absorb part of the stresses. Because some fibers are more deformable, they collaborated during the opening and propagation of cracks. After cracking, the composite stiffness decreased.

Therefore, it is suitable for the purpose of manufacturing hollow blocks for masonry, according to the NBR 6136 [46] where the minimum compressive strength is 8.00 MPa, and in the manufacture of slabs and tiles in which the samples reached flexural strength values above 4.00 MPa, the minimum value specified by EN 12,467 [47] for flat fiber cement sheets.

## Figures and Tables

**Table 1:** Chemical and physical composition of ordinary Portland cement, fly ash and metakaolin.

Materials	L.O.I (%)	CaO (%)	SiO_2_ (%)	MgO (%)	Al_2_O_3_ (%)	SO_3_ (%)	Fe_2_O_3_ (%)	K_2_O (%)	TiO_2_ (%)	Specific Gravity (g/cm^3^)	Fineness (cm^2^/g)
Cement (OPC)	5.38	54.5	19.96	8.34	4.25	3.81	2.29	0.92	0.26	3.17	3605
Fly ash (FA)	0.8	2.31	61.18	0.70	30.64	0.36	1.67	1.27	0.81	2.17	3337
Metakaolin (MK)	2.38	0.12	43.90	-	46.56	0.08	3.05	0.46	0.76	2.81	12,531

L.O.I: loss on ignition.

**Table 2:** Chemical composition of untreated fibers.

Fiber	Holocellulose (%)	Alpha-Cellulose (%)	Hemicellulose (%)	Lignin (%)	Extractives (%)
Piassava	53.16	51.45	1.71	45.68	1.17
Tucum palm	78.90	77.52	1.38	17.36	3.74
Razor grass	95.3	86.85	8.45	0.81	3.89
Jute	83.18	64.07	19.11	12.46	4.36

**Table 3:** Pastes compositions.

Pastes	Cement (OPC) (g)	Metakaolin (MK) (g)	Fly Ash (FA) (g)	Water (g)	S.P (%)
REF (control)	500	0	0	200	-
10MK + 40FA	250	50	200	200	-
20MK + 30FA	250	100	150	200	0.30
30MK + 20FA	250	150	100	200	0.30
40MK + 10FA	250	200	50	200	0.50

**Table 4:** Percentage of CH and 28d-compressive strength of cement pastes.

Cement Pastes	REF (100% OPC)	10 MK + 40 FA	20 MK + 30 FA	30 MK + 20 FA	40 MK + 10 FA
CH%	16.86	6.66	5.03	4.13	3.84
Compressive Strength (MPa)	36.92	27.82	37.89	39.40	42.12

**Table 5:** Crystallinity index (CI %) and compressive strength (CS) of mortars with untreated and treated fibers.

Fiber/Cure	% Fiber	Treatment	CI (%)	CS (MPa)
Piassava/water	1.50	UT	39.83	28.78
		T	51.8	26.71
Piassava/water	3.00	UT	39.83	26.14
		T	51.8	25.12
Piassava/water	4.50	UT	39.83	25.65
		T	51.8	23.71
Piassava/CO_2_	1.50	UT	39.83	24.73
		T	51.8	22.94
Piassava/CO_2_	3.00	UT	39.83	21.86
		T	51.8	20.61
Piassava/CO_2_	4.50	UT	39.83	20.45
		T	51.8	22.97
Tucum palm/water	1.50	UT	59.84	23.29
		T	66.73	20.84
Tucum palm/Water	3.00	UT	59.84	20.87
		T	66.73	19.65
Tucum palm/water	4.50	UT	59.84	21.21
		T	66.73	14.59
Tucum palm/CO_2_	1.50	UT	59.84	18.27
		T	66.73	19.32
Tucum palm/CO_2_	3.00	UT	59.84	16.19
		T	66.73	18.41
Tucum palm/CO_2_	4.50	UT	59.84	14.51
		T	66.73	22.03
Razor grass/water	1.50	UT	60.23	26.02
		T	55.21	25.48
Razor grass/water	3.00	UT	60.23	24.98
		T	55.21	24.36
Razor grass/water	4.50	UT	60.23	27.72
		T	55.21	22.12
Razor grass/CO_2_	1.50	UT	60.23	18.00
		T	55.21	18.06
Razor grass/CO_2_	3.00	UT	60.23	19.17
		T	55.21	20.19
Razor grass/CO_2_	4.50	UT	60.23	15.91
		T	55.21	20.09
Jute/water	1.50	UT	49.28	23.04
		T	57.53	20.34
Jute/water	3.00	UT	49.28	15.88
		T	57.53	18.66
Jute/water	4.50	UT	49.28	17.43
		T	57.53	16.64
Jute/CO_2_	1.50	UT	49.28	14.97
		T	57.53	22.01
Jute/CO_2_	3.00	UT	49.28	13.84
		T	57.53	15.43
Jute/CO_2_	4.50	UT	49.28	13.33
		T	57.53	19.19

UT—untreated fibers; T—treated fibers.

## Data Availability

The data presented in this study are available on request from the corresponding author.
